# Common and Distinct Functional Brain Networks for Intuitive and Deliberate Decision Making

**DOI:** 10.3390/brainsci9070174

**Published:** 2019-07-20

**Authors:** Burak Erdeniz, John Done

**Affiliations:** 1 Department of Psychology, İzmir University of Economics, 35330 Izmir, Turkey; 2 Department of Psychology and Sports Sciences, School of Life and Medical Sciences, University of Hertfordshire, Hatfield AL 10 9AB, UK

**Keywords:** intuitive decision, deliberate decision, striatum, novelty, automated cognition, reinforcement learning, fMRI

## Abstract

Reinforcement learning studies in rodents and primates demonstrate that goal-directed and habitual choice behaviors are mediated through different fronto-striatal systems, but the evidence is less clear in humans. In this study, functional magnetic resonance imaging (fMRI) data were collected whilst participants (*n* = 20) performed a conditional associative learning task in which blocks of novel conditional stimuli (CS) required a deliberate choice, and blocks of familiar CS required an intuitive choice. Using standard subtraction analysis for fMRI event-related designs, activation shifted from the dorso-fronto-parietal network, which involves dorsolateral prefrontal cortex (DLPFC) for deliberate choice of novel CS, to ventro-medial frontal (VMPFC) and anterior cingulate cortex for intuitive choice of familiar CS. Supporting this finding, psycho-physiological interaction (PPI) analysis, using the peak active areas within the PFC for novel and familiar CS as seed regions, showed functional coupling between caudate and DLPFC when processing novel CS and VMPFC when processing familiar CS. These findings demonstrate separable systems for deliberate and intuitive processing, which is in keeping with rodent and primate reinforcement learning studies, although in humans they operate in a dynamic, possibly synergistic, manner particularly at the level of the striatum.

## 1. Introduction

A large body of functional imaging research has explored the neural mechanisms involved in value-based decision making (for reviews see [1,2,3,4]). Despite reliable findings, it is unclear whether there are different neural systems for deliberate, as opposed to intuitive, decision making in humans or, using the cognitive neuroscience vernacular, for goal-based versus habitual choice [5]. In the psychological literature, deliberate decision making occurs when making a choice between unfamiliar options whose outcomes are unknown in a context where there is a high degree of outcome uncertainty [6,7]. Deliberation occurs when computing the contingency or causal relationship between conditional stimulus (CS), response (R), and their associated outcome (O) [8,9]. This requires a cognitive ‘scaffold’ composed of problem-relevant algorithms [10,11], rules, and mental models [12] in order to solve the credit assignment problem [13]. 

Intuitive decision making occurs once the contingencies between CS, R, and O are well-learnt, such that probabilities of outcomes and their value become integrated dimensions of the CS and are, hence, available at the point of stimulus perception (stimulus-elicited) [14,15,16,17,18]. It remains unclear whether deliberate and intuitive decision making involve separate cognitive processes [19] or a common set of rule-based cognitive processes, with a shift toward more efficient associative processing during intuitive decision making [20,21,22].

Neuroscience research in rodents and primates supports separate fronto-striatal routes used to estimate the value of options in deliberate, or ‘goal-based’, as opposed to intuitive-based, or ‘habit’, decision making (for reviews see [1,5,16,23]). These fronto-striatal systems comprise the ‘associative’ (dorsomedial striatum) and ‘sensorimotor’ (dorsolateral striatum) fronto-striatal routes for goal-based and habitual-based valuation, respectively [1,23].

While some functional neuroimaging studies with humans used reinforcement learning tasks to examine separate valuation systems (for a review see, [5]), the majority of such studies have used many trials (often in the hundreds), thus confounding early goal-based learning with later habitual or what we here refer to as ‘stimulus-elicited’ choice [24,25,26,27,28]. Some studies comparing early vs. late learning, or used pre-training, have shown reduced involvement of associative striatum with learning [29,30,31]. Few studies have ensured over-learning of the CS-outcome pairings [32,33], and these have demonstrated that with overlearning there is a shift from associative to the sensorimotor striatum for CS valuation. However, these studies involved reinforcement learning of either a variable interval schedule [32] or learning a sequence of choices [33], both of which are particularly suited to inducing habitual behavior [23,25,34,35]. Ashby et al. [36] argued that such a shift is limited to these reinforcement learning paradigms alone, and they will not be found in the more commonly used conditional associative learning (CAL) or monetary incentive learning paradigms, in which participants learn to discriminate between two or more CS to maximize reward. One such study designed to eliminate all learning through the use of highly overlearnt options (playing cards) reported that the sensori-motor route was used in value processing of conditioned stimuli [37]. However, both circuits may be operating in parallel in most decision-making situations [20,23], switching during the course of the given task [5,38,39]. This would create a methodological problem for imaging studies in which the analysis involves averaging across trials of different event types (e.g., goal vs. stimulus-elicited trials) and then comparing them with subtraction analysis. 

The primary aim of the current study was to evaluate whether the associative processing route is used predominantly for deliberate choices, whereas the sensori-motor route is used predominantly for stimulus elicited-valuation (familiar choices), using a CAL paradigm. A single set of CS were used with consistent reward probabilities, which had been extensively trained before the imaging session [24]. Furthermore, behavioral criteria (e.g., response time), which is no further decrease in response times after the pre-training session, were used to validate whether participants demonstrated either goal-based or stimulus-elicited processing [27,40]. An additional challenge ignored in previous studies, (notably [29] and [41]) is the increase in the blood oxygenation level dependent (BOLD) signal across most brain regions during goal-based cognition [42]. This is thought to arise either due to a global increase in the brain’s BOLD signal during effortful cognition or in response to greater attention, which increases BOLD signal, particularly in primary sensory cortices [43,44]. Alternatively, the decrease in BOLD signal when processing familiar stimuli might result from a shift from coarse neural coding during early learning to fine coding for familiar stimuli [42,45]. All such explanations would result in a reduction in the average BOLD signal across all clusters of voxels for familiar stimuli and, hence, represent a potential confound. Kelly and Garavan [42], therefore, recommend that traditional contrast analysis be compared with a psycho-physiological interaction (PPI) analysis to explore the potential reorganization of functional connectivity resulting from a change from goal-based processing to stimulus-elicited processing. 

We hypothesized that the ubiquitous increase in global BOLD signal during all deliberate cognition should be included as a functional imaging signature for goal-based decision making and supplement the behavioral criteria described by Seger and Spiering [40]. Thus, deliberate, but not intuitive, valuation should be associated with high levels of activation in the dorso-fronto-parietal system, together with increased activation of the sensory cortex [46,47,48,49].

## 2. Materials and Methods

### 2.1. Participants

Twenty right-handed healthy volunteers (12 male, 7 female; mean age 25, range: 24–32) were recruited, but only 19 participants (12 male, 6 female) were included in the analysis. One participant was excluded from the analysis due to insufficient behavioral performance; for instance, this participant showed no learning effect and informed the experimenter that she was making random choices in all conditions. All of the participants were: i) pre-assessed to exclude those with a prior history of neurological and psychiatric illness, ii) provided with written and verbal instructions about the experiment before the functional magnetic resonance imaging (fMRI) session, and iii) debriefed after the experimental session and paid £20 for participating. All subjects gave their informed consent for inclusion before they participated. The study was conducted in accordance with the Declaration of Helsinki, and the protocol was approved by the Ethics Committee of Bedfordshire NHS Research Ethics Committee (Decision Number: 06/Q0202/21).

### 2.2. Experimental Design

This experiment comprised two parts: the pre-training session (for familiarization with the stimuli) was designed to ensure learning of a set of stimulus pairs (*k* = 12) and was conducted outside of the scanner, while the main experimental session involved the collection of fMRI data and included presentation of both the familiar stimulus pairs used in the pre-training session as well as a set of previously unseen novel stimulus pairs (*k* = 12). E-prime software (www.psychologysoftware.com) was used to control the presentation of stimuli and collect the response data for both the pre-training and main experimental sessions.

#### 2.2.1. Pre-Training

**Figure 1:**
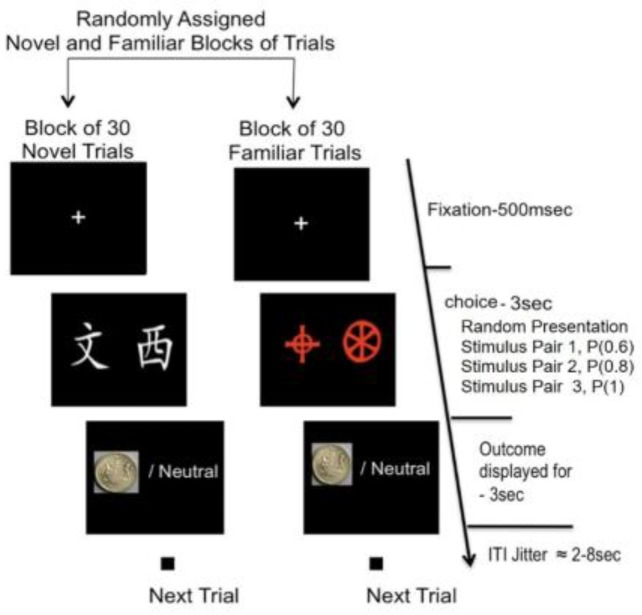
Probabilistic learning task used during pre-training and scanning sessions. Subjects were presented with two abstract visual stimuli (either familiar or novel pair). Subjects selected one of the stimuli, and their choice was followed by a probabilistic feedback on the screen displaying either a pound picture or written “neutral”.

Figure 1 presents a diagram showing the structure of a single trial, with the training task involving 240 such trials. Each trial began with a fixation cross of 500 ms presented in the middle of an LCD screen, followed by the presentation of one pair of stimuli for approximately 3 s. During those 3 s, participants were asked to respond by choosing one of two response keys to indicate their preferred stimuli. After making their choice, feedback was immediately presented on the screen for about 3 s. For each correct prediction, the rewarding feedback was a large image of a £1 coin, and for non-rewarded feedback the word ‘neutral’ was displayed on the screen. Subjects were informed that the amount of money they would receive at the end of the study would be contingent on the money accumulated after completion of both the pre-training session and main experiment, and that this amount will be multiplied by a probability. However, in order to avoid disadvantaging any participant, 20 pounds was awarded to all participants on completion of the main experiment. Twelve stimulus pairs were used in the pre-training session with each stimulus pair composed of Chinese or Agathadaimon font. The pairs of stimuli were counterbalanced across participants so that each was trained with only one type of font.

Stimulus pairs differed in their uncertainty of reward outcome. For high-outcome uncertainty pairs, the probability of reward was 0.6 for one CS (i.e., one of the two options) and with corresponding probability of not being rewarded of 0.4. For mid-outcome uncertainty pairs, the probability of reward for one CS was 0.8, and for the other CS, 0.2. Finally, the last stimulus category was deterministic rather than probabilistic; the outcome was certain if the participant chose the correct CS, resulting in a reward every time, whereas the other CS would always result in non-reward. Each stimulus pair was presented equally often over the 240 trials (i.e., 20 exposures for each pair). Subjects were fully instructed about trial structure and the objective of maximizing the reward but not the nature of the stimulus-reward (S–R) contingencies. All responses were made on an MRI-compatible Lumina response box, which comprised four key pads, but only the two at the right end were used, so that the subject could position their index and middle fingers over the keys and, thus, minimize hand movements. Choice of the CS on the left side, as opposed to right, of the screen was indicated by pressing the index (left-most) finger, as opposed to the middle (rightmost) finger of their right hand, respectively, and vice versa, thereby ensuring a high degree of S–R compatibility [50]. 

#### 2.2.2. Main Experiment

The main experiment was conducted with the participant in the scanner. A total of 240 trials were presented using the same format for each trial that had been used previously in the pre-training session (Figure 1) apart from the introduction of a jitter delay between trials. Therefore, during the main experiment, participants were presented with 120 novel and 120 familiar stimulus trials. There were four separate sessions, comprising 60 trials in each (240 trials/4 session), separated by a 2 min rest interval. Each session was split into further two blocks with one block comprising novel stimulus pairs only (3 pairs randomly presented 10 times each, making 30 trials) and the other, familiar stimulus pairs only (a block comprised 30 trials made up of 3 pairs, randomly presented 10 times) (see Figure 1). The familiar stimulus pairs were those used in the pre-training session (outside the scanner). Each novel CS pair was composed of the character set not used previously in the pre-training session; therefore, both the character set and the stimulus pairs themselves were novel. However, each pair was either a low/moderate/certain probability of outcome pair corresponding to the 3 probabilistic categories. After each block of 30 novel trials, the set of 3 stimulus pairs was replaced by another set of 3 pairs to ensure novelty across all 4 sessions. The blocking of novel and familiar stimulus pairs was intended to maximize the effect of “framing” [51], that is, to ensure that participants adopted a ‘goal-directed’ or ‘stimulus-elicited’ mental set for processing novel and familiar stimulus pairs, respectively. This also prevented activity-related contextual novelty of temporal unexpectancy (i.e., unsure which type of stimulus pair would come next). This type of novelty (contextual) has often been noted in tasks resembling the oddball paradigm [52]. At the end of each trial in the main experiment, there was a random inter-trial interval of 2–8 s (random jitter) in order to separate the rewarding outcome and stimulus presentation in the next trial.

### 2.3. Functional Magnetic Resonance Imaging

#### 2.3.1. Image Acquisition

Functional imaging was conducted using 3-Tesla Siemens Magnetom MRI scanner to acquire gradient echo T2* weighted echo-planar (EPI) images with BOLD (blood oxygenation level dependent) signal contrast (3 × 3 × 3 mm voxel size). Imaging parameters were optimized to minimize signal dropout in medial ventral prefrontal and anterior ventral striatum: we used a tilted acquisition sequence at 30° to the AC–PC line [53]. Each volume comprised 36 axial slices of 3 mm thickness and 3 mm in-plane resolution with a TR time (repetition time) of 3 s. The flip angle was 90 degrees. T1 weighted structural images (1 × 1 × 1 mm voxel size) were also acquired for each participant. Head movement was minimized by padding the participants’ heads.

#### 2.3.2. Image Analysis

Image analysis was performed using statistical parametric mapping SPM8 software (Wellcome Department of Imaging Neuroscience, Institute of Neurology, London, United Kingdom). For all participants, the images were realigned according to the first volume, in order to correct for motion in the scanner, and their functional EPI images were co-registered to anatomical images and normalized to a standard Montreal Neurological Institute (MNI) template. Spatial smoothing was applied using a Gaussian kernel with a full width at half-maximum (FWHM) of 8 mm for each participant’s data. Statistical analyses were performed on each participant’s data using the general linear model in SPM8. The fMRI time series data were modeled by a series of events, convolved with a canonical haemodynamic response function (HRF). The general linear model (GLM) included one regressor for each stimulus type (novel vs. familiar) and each level of probability of reward for a correct choice (i.e., 0.6, 0.8, 1.0) and included six motion regressors. The second level of the analysis involved voxel-wise comparisons across subjects analysis of variance (ANOVA), which were computed from the single subjects’ contrast images, treating each subject as a random effect. Coordinates of significant local maxima are reported in a standard stereotaxic reference space (MNI, Montreal Neurological Institute), and group functional overlays are displayed on a single subjects’ anatomical scans.

We also conducted hypothesis-driven statistical analyses, namely PPI analyses, to evaluate whether there was a shift from the associative (goal-based) to sensori-motor (stimulus-elicited) circuits in striatum for novel and familiar CS, respectively. PPI assesses how the activity within one brain region or network of regions is coupled with a predefined region of interest (seed region). The rationale for using PPI analysis in the current study was to detect whether there was a coupling between the different striatal nuclei and other regions, particularly ‘seed regions’ in the prefrontal cortex, that differentiated goal-based processing from stimulus-elicited processing, derived from the subtraction analyses. Finally, we reported both family wise error (FEW) corrected and uncorrected results. For the uncorrected results, a minimum cluster size of 10 voxels was used, unless otherwise mentioned [54].

## 3. Results

### 3.1. Behavioral Results

**Figure 2:**
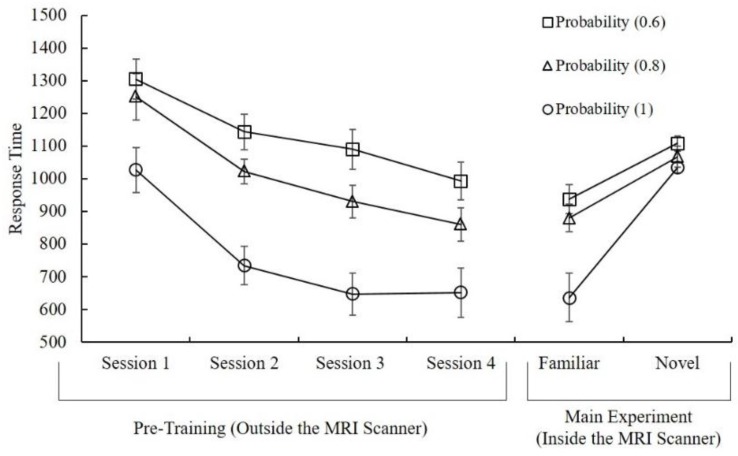
Response times for the pre-training (Session 1, 2, 3, and 4) and main experiment showed for all outcome probabilities. Error bars indicate standard deviations.

In order to compare the response times (RTs) for novel vs. familiar trials, we first performed a 3 (probability 0.6, 0.8, 1) × 5 (Session 1, 2, 3, 4, Familiar) repeated measures ANOVA. Results revealed that the main effect of session was statistically significant (F (4, 72) = 35.98, *p* < 0.001, partial η2 = 0.67). Pairwise comparisons showed that response times of participants decreased over the sessions (F (1, 18) = 35.98, *p* < 0.001 (session 1 to session 2); F (1, 18) = 11.66, *p* = 0.003 (session 2 to session 3); and F (1, 18) = 5.06, *p* = 0.037 (session 3 to session 4)). Comparisons also revealed that there was no significant difference between session 4 (pre-training) and familiar condition (MRI session) (*p* > 0.05) after adjustment for multiple comparisons. This indicates that no further learning-related changes occurred in response times, which applies to both the familiar pairs during the fMRI session in the main experiment and session 4 (last pre-training session), which was outside the scanner (Figure 2). For similar learning criteria that indicate automaticity, please see [24]. Furthermore, the main effect of probability was also statistically significant (F (2, 36) = 50.18, *p* < 0.001, partial η2 = 0.74). Pairwise comparisons revealed that the response time decreased significantly when the probability increased (F (1, 18) = 69.35, *p* < 0.001 (from 0.6 to 0.8); F (1, 18) = 41.75, *p* < 0.001 (from 0.8 to 1); Bonferroni corrected). Finally, the interaction effect of probability and session did not reach significance level (F (8, 144) = 0.37, *p* > 0.05) (see Figure 2).

Secondly, a 2 (novel vs. familiar) × 3 (probability) repeated measures ANOVA was conducted in order to compare RTs during the imaging session. Familiar trials showed shorter response times compared to novel trials (F (1, 18) = 35.15, *p* < 0.001, partial η2 = 0.66). The main effect of probability was also statistically significant (F (2, 36) = 23.73, *p* < 0.001, partial η2 = 0.57). Pairwise comparisons revealed that participants responded slower in 0.6 probability than 0.8 (F (1, 18) = 39.25, *p* < 0.001). Also, they responded slower in 0.8 than 1 (F (1, 18) = 16.02, *p* < 0.001) after adjustments for multiple comparisons, Bonferroni corrected. Finally, the interaction effect was statistically significant (F (2, 36) = 12.82, *p* < 0.001, partial η2 = 0.42) between probability and stimulus familiarity. 

**Figure 3:**
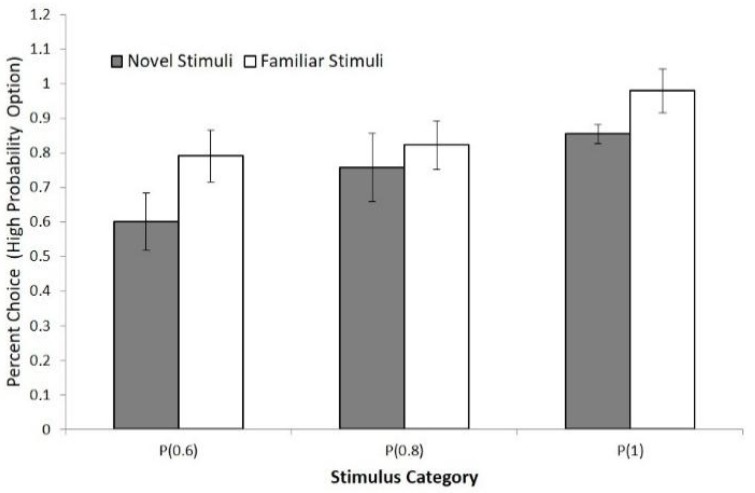
Percent choices for high probability winning option for the novel and familiar stimuli obtained during the main experiment, collapsed across all outcome probabilities. Error bars indicate standard deviations.

Additionally, for the main experiment during the fMRI session, in order to examine the differences in behavioral learning performance for different stimulus pairs, we calculated the percentage of choice for each probability condition (0.6, 0.8, 1) and for each stimulus category (novel and familiar) (Figure 3). For example, if the participants learn to choose the winning option for the (0.6) pair, they will allocate at least 60% their responses to that option [55], sometimes referred to as the matching strategy, which is an indication that learning has reached asymptotic levels [56]. In order to measure the learning performance between the novel and familiar stimulus sets during the fMRI session, we compared the number of percent choice responses for each stimulus pair for both novel and familiar conditions (see Figure 3). This comparison used a 2 (novel, familiar) × 3 (probability 0.6, 0.8, 1) repeated measures ANOVA conducted on the percentage of choice data (high-probability option). Results revealed that in the familiar condition, participants chose the high-probability winning option more than in the novel condition (F (1, 18) = 22.73, *p* < 0.001, partial η2 = 0.57). Furthermore, the main effect of probability was also statistically significant (F (2, 35) = 19.27, *p* < 0.001, partial η2 = 0.53). Pairwise comparisons revealed that accuracy increased with probability (F (1, 18) = 4.68, *p* = 0.045 (between 0.6 and 0.8); F (1, 18) = 14.63, *p* < 0.001 (between 0.8 and 1)) after adjustments for multiple comparisons. Finally, the interaction effect did not reach significance level (F (2, 34) = 2.26, *p* > 0.05). Overall, these results provide additional support for the notion that, in addition to the response time data, the behavioral data for the percent choice show significant differences for novel and familiar stimulus pairs.

### 3.2. Imaging Results

The full network of regions activated across both novel and familiar stimulus types was investigated using a standard analysis of variance (ANOVA) implemented in SPM8. There were two main effects, namely CS type with two levels (novel CS and familiar CS) and probability of reward with three levels (0.6, 0.8, 1), together with their interaction. The overall mean for all trials (i.e., conjunction analysis for all CS vs. baseline) manifested a widespread network of regions. Of particular note is the high levels of activation throughout the striatum (including both ventral and dorsal regions), medial frontal regions and orbito-frontal cortex (OFC), cingulate cortex, hippocampus, lateral parietal cortices, precuneus, and cerebellum (*p* < 0.05, FWE corrected). For a full list of activated regions, please refer to Table 1. 

Furthermore, the highly significant main effect of CS type in the ANOVA resulted from the novel CS, producing a significantly greater activation across numerous brain regions. This fits with the expectation, described in the introduction, of a greater BOLD signal for novel stimuli, or goal-directed processing [42]. The significant main effect of stimulus probability was a result of bilateral clusters that included inferior frontal gyrus, and putamen. In the right hemisphere, activation extended into the right caudate. The clusters and coordinates for peak voxel for the main effect of reward probability and the interaction (probability × stimulus type) are reported in Table 2.

**Figure 4:**
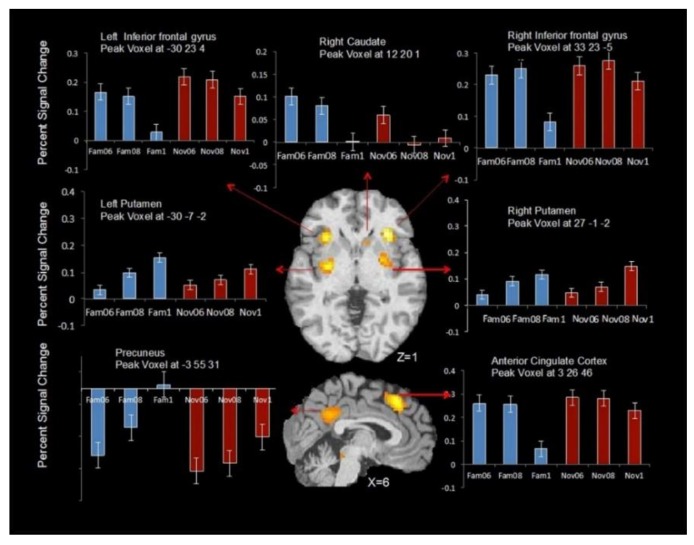
Areas in the bilateral putamen, bilateral inferior frontal gyrus, right caudate, precuneus, and anterior cingulate cortex showing uncertainty-related activity for CS regardless of whether the stimulus is novel or familiar (*p* < 0.001, a minimum cluster size (*k*) = 10 voxels).

Post hoc analyses of the main effect of outcome probability showed that activation in the right caudate was associated with decreased expectation of reward outcome (i.e., decrease with outcome probability), whereas activation in the left and right putamen was associated with increased expectation of reward outcome (see Figure 4). The right and left inferior frontal gyrus also showed a decrease in activation with increasing certainty of reward.

**Figure 5:**
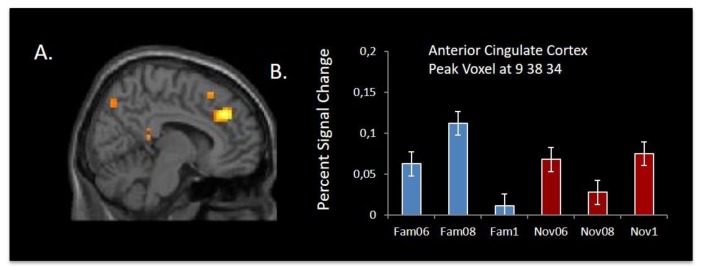
(**A**) Interaction contrast overlaid on medial frontal gyrus showing significant interaction between uncertainty and novelty (*p* < 0.001, a minimum cluster size (k) = 10 voxels). (**B**) Percent signal change for the peak voxel in the anterior cingulate gyrus.

Post hoc analysis of the probability × stimulus type interaction revealed a large cluster in the right anterior cingulate, including brodmann area (BA) 32, extending into medial frontal cortex (*p* < 0.001, uncorrected) (Figure 5). This interaction effect was due to significant activations above baseline for all conditions except for the certain condition (probability = 1:0) for the familiar stimuli. 

### 3.3. Subtraction Analyses—Novel vs. Familiar Stimuli

**Figure 6:**
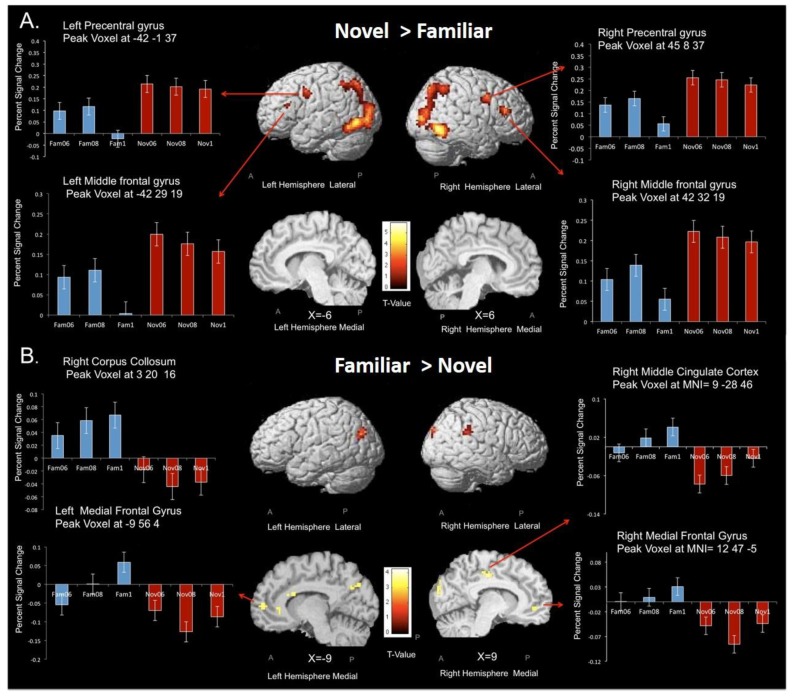
Regions demonstrating greater activation for the (**A**) novel CS > familiar CS contrast across all eighteen participants (*p* < 0.001 uncorrected, minimum cluster size 10 voxels). For the two top images, activation is overlaid on the lateral surfaces of the cortex separately for each hemisphere (the image on the right side of the figure depicts the right hemisphere whereas the image on the left side of the figure depicts the left hemisphere). The bottom two images show activation in the medial surface of the cortex. (**B**) Shows the regions demonstrating greater activation for the familiar CS > novel CS contrast across all participants (*p* < 0.001, uncorrected, minimum cluster size 10 voxels). For the two images, the top activation is overlaid on the lateral surfaces of the cortex separately for each hemisphere, for both figure (**A**,**B**), the color bar represents the t-value where yellow regions correspond to higher t-values and a larger effect size. The bar graphs on the sides of each figure depict percent signal change for the novel and familiar CS that were shown for the peak voxels.

Dissociating the neural systems for (i) goal-based and (ii) stimulus-elicited decision making was achieved by comparing the contrasts of novel CS > familiar CS and familiar CS > novel CS. Firstly, we wanted to examine whether the functional imaging criteria validated whether goal-based processing predominated for choices with the set of novel CS, rather than familiar CS (Table 3 and Table 4 and Figure 6 present the data for both contrasts). With regard to the novel CS > familiar CS contrast analysis, greater dorsal fronto-parietal network involvement was observed for this contrast; greater activity was found along the intraparietal sulcus, extending into inferior and superior parietal lobes, and there was greater activation in dorsal frontal areas, particularly along the middle frontal gyrus, to include DLPFC (middle frontal gyrus BA46) in both left and right hemispheres (Table 3). These regions define the dorso-parietal network [46,57]. This contrast also revealed more widespread and increased activation for novel CS in superior, medial, and inferior occipital gyri (Table 3). Both results meet the fMRI criteria outlined in the introduction for distinguishing goal-based and stimulus-elicited decision making.

Our main research question was whether the associative and sensori-motor fronto-striatal circuits mediated goal-directed and stimulus-elicited choices. In particular, we assumed there should be a dissociation in both frontal and striatal regions. Under this assumption, goal-based decision making would show increased activation in the fronto-parietal system, involving DLPFC, but, in addition, we would expect to see greater involvement of the head and possibly the body of the caudate (ventromedial striatum) for novel CS and a greater involvement of the putamen with familiar CS. 

A dissociation in the prefrontal cortex was observed (see Figure 6). Greater activation in bilateral middle frontal gyrus (BA 46) was found in the novel CS > familiar CS contrast, and greater activation was found in bilateral VMPFC (BA 10) for the familiar CS > novel CS contrast, presumably due to VMPFC being able to code predicted outcome values, which, in this study, equate to expected values for each CS (Table 4). However, it should be noted that this finding largely is due to bilateral deactivation in this region for novel CS (see bar chart for left and right medial frontal gyri in the lower panel of Figure 6). Outcome probability was bilaterally coded in the left VMPFC for familiar CS but not novel CS (see Figure 6). Neither the novel CS > familiar CS contrast, nor familiar CS > novel CS contrast, revealed any differential striatal activation in the left or right head nor in the body and tail of the caudate or putamen. 

### 3.4. Psycho-Physiological Interaction Analysis

**Figure 7:**
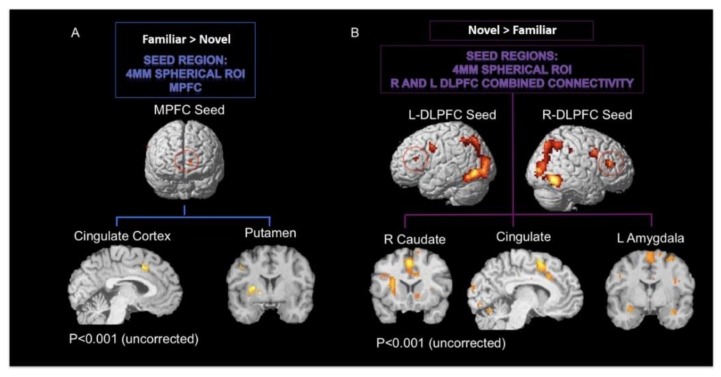
Regions demonstrating significant connectivity for the (**A**) familiar CS > novel CS and (**B**) novel CS > familiar CS contrasts.

The psychophysiological interaction analysis (PPI) permits examination of those brain regions showing enhanced coupling with a predefined region of interest (seed region) given the psychological condition (i.e., novel CS > familiar CS or familiar CS > novel CS) [58]. Thus, we selected the seed regions in the prefrontal cortex identified in the novel CS > familiar CS contrast, (bilateral middle frontal gyrus left MNI −42, 29, 19; right MNI 42, 32, 19), and the familiar CS > novel CS contrast, (right medial frontal gyrus MNI 12, 47, −5), and we evaluated the activity of brain regions modulated by activity in these two frontal regions (Figure 7).

In order to perform a PPI analysis at the single subject level, the BOLD signal was extracted from seed regions for each participant. These were defined as 4 mm spherical region of interest (ROI) around each participant’s peak voxel (individual contrast thresholds for determining seed regions, see below). Physiological activity of seed regions was extracted as the first principal component of the time series of all active voxels within a 4 mm radius sphere, centered on the most significant voxel within the ROI. Significance of the voxels was based on the contrast specified for each participant (*p* < 0.005, uncorrected). Subjects were excluded from the analysis when there were no significant voxels in this statistical threshold. Based on this exclusion criterion for both PPI analyses, 14 out of 19 participants were included. The PPI term was created for each participant by multiplying the deconvolved and mean-corrected BOLD signal with the psychological vector. After convolution with the HRF, mean correction, and orthogonalization, the three regressors (PPI term, physiological vector, and psychological vector) were entered into the statistical analysis to determine condition-dependent changes of functional connectivity over and above any main effect of task or any main effect of activity in the corresponding brain areas. In the PPI contrasts, the PPI term was computed against an implicit baseline. Random-effects analyses were performed on single-subject PPI contrast images (*p* < 0.001, uncorrected) and carried to group analysis. 

The PPI analysis with prefrontal seed regions derived from the novel CS > familiar CS contrast, namely BA46, revealed co-variations with bilateral dorsomedial (caudate) striatal activity and right ventral striatum (putamen) as well as with the amygdala, insula, and several small clusters in the lateral prefrontal cortex and cingulate gyrus (see Figure 7, Table 5).

The PPI analysis using the medial prefrontal cortex seed region, derived from the familiar CS > novel CS contrast, resulted in co-activation with the dorsolateral striatum (putamen) bilaterally, with the cingulate cortex, and additional activation occurs mainly in the medial surface of the frontal cortex (Table 6). Finally, it is important to note the PPI results are reported uncorrected at *p* < 0.001 and do not survive family-wise error correction. The coordinates for these comparisons are reported in Table 5 and Table 6.

## 4. Discussion

The principle aim of the current study was to find out whether reward anticipation during deliberate and intuitive decision making involved separate fronto-striatal systems, namely the ‘associative’ and ‘sensori-motor’ fronto-striatal systems, respectively. The behavioral and functional imaging criteria for goal-based processing were met for the trials that involved novel CS but not familiar CS. During learning, response times reduced significantly for the novel CS, and a pattern of significantly greater BOLD activity was observed across numerous brain regions, most notably in the occipital cortex. Furthermore, the fronto-parietal network was more active in novel trials relative to familiar trials for novel CS only. In addition, there was a significant activation in the temporo-parietal regions. In the literature, previous animal and human studies showed that medial temporal lobe (MTL) structures often respond to stimulus repetition that shows reduction of neural activity with repeated stimulus presentation [59,60]. It was suggested that this widely distributed network of temporal and parietal regions is involved in the novelty/familiarity effects that may be responsible for learning visual associations [61,62,63,64].

Greater activation for familiar CS relative to novel CS occurred in a small number of brain regions, including VMPFC and posterior cingulate, in line with most other studies comparing goal-based and automated cognitive processing [42]. No striatal areas showed a higher level of activation for familiar relative to novel CS. However, we found increased activation in the same bilateral region of VMPFC for familiar CS, as reported in a number of other studies [10,65,66,67,68]. A linear increase in activation in the VMPFC was also detected with increased probability of reward (equivalent to expected value) for the familiar CS only (see Figure 6B). This same region of VMPFC showed significant deactivation during the novel CS trials. Tanaka et al. [69] also reported an increasing deactivation in VMPFC in proportion to the amount of goal-based processing during an instrumental reinforcement learning task. fMRI studies of self-control in dieting [70] or addiction [71] have also reported deactivations in this region when implementing a top-down strategy to inhibit prepotent responses. Furthermore, functional connectivity between VMPFC and DLPFC has been reported in other studies [72,73,74]. This finding supports the dual-route view of decision making in so far as VMPFC activations occur after the CS-O contingency is learnt, whereas deactivation occurs when the CS value is unknown. An alternative interpretation is that VMPFC activity reflects an increased expectation of reward, or increased decision confidence in the familiar blocks, because subjects might be more confident of making the correct choice [75]. 

Our second main prediction was that novel CS would engage the dorsomedial striatum (caudate head and possibly body), whereas value processing of familiar CS would engage the dorsolateral striatum (putamen). This was not observed for any of the contrast analyses, even with a liberal threshold for statistical significance. This would suggest that, unlike the frontal cortex, there is no manifest shift from dorsomedial to dorsolateral processing in the striatum for value-based decision making, using conditional associative learning paradigms. Notwithstanding this finding, the PPI analysis demonstrated functional connectivity between the VMPFC seed region and the dorsolateral striatum (putamen) as well as separate functional connectivity between the DLPFC and dorsomedial striatum (caudate). These results, therefore, support the view that there is some anatomical and functional dissociation between goal-based and stimulus-elicited decision making in the striatum [76]. The findings between the PPI and contrast analyses can be perhaps best interpreted as showing that decision making systems have a dynamic relationship during conditional associative learning type tasks. Until recently, the view has been of these two systems working in parallel but in competition [4,38]. More recent thinking is that these two systems operate synergistically, with heuristics being used as and when appropriate [77], for example, when the cognitive load is high [78] or at different points throughout the series of steps in a typical decision-making process [5,39]. 

Such a dynamic relationship between the different striatal regions might be more apparent in CAL tasks, whereas in variable intervals or sequence RL tasks, a less dynamic relationship may occur, such that processing is based predominantly in one system according to the degree of learning. Ashby et al. [36] argued that these two experimental paradigms are particularly susceptible to becoming habitual with overlearning.

An alternative account is that any activation due to action selection is included in the conjunction analysis and discounted in contrast analyses, since the same ‘go left’/’go right’ action selection is made for both sets of CS when choosing the left side/right side stimulus, respectively. Thus, the transition from caudate, for goal-directed processing, to putamen, for habitual processing, may be more apparent when learning action outcomes rather than stimulus outcomes [79,80,81,82,83]. However, the processing of stimulus values in VMPFC is well established [68,84], which would account for the significant VMPFC activation in the familiar CS > novel CS contrast. Such an explanation presumes a predominantly action selection or motor execution role for the caudate and putamen, when there is considerable evidence for the caudate being involved in numerous other cognitive functions [see 74] including working memory [85], reasoning [86], category learning [87,88], reward prediction, and stimulus anticipation [89]. Therefore, further investigation is needed in deducing that the contrasting activation in the striatum for conjunction and contrast analyses is best explained by valuation of actions rather than stimuli. The results also suggest that the striatum is involved at a very early stage of learning CS values since the right caudate coded uncertainty of rewarding outcomes for novel CS, whereas the putamen coded for the converse, namely reward probability for novel CS. Such early learning of outcome probabilities was not evident in any region of the PFC. 

The finding of striatal coding of reward probability has been reported in many studies [5,20,28,37,88], but not all [26]. In addition, faster learning of CS-R-O associations in caudate than in PFC has been previously reported by others [31,89,90]. It has been suggested that the striatum rapidly learns a small set of associations and trains the slower learning PFC [36,90]. If the striatum represents the outcome probabilities, or expected values for a small set of contextually relevant stimuli, then one might expect a similar coding of expected values for novel and familiar CS alike, as observed in the current study. The large cluster involving the right anterior cingulate cortex (ACC), extending dorsally into adjacent cortical regions (BA 32, with some extension into inferior BA6), which also coded for outcome probability for familiar CS, fits with the meta-analysis reported by Volz et al. [91]. This region of the anterior cingulate also approximates to the ROI 5 in the meta-analysis of Torta and Cauda [92], who noted that this region is active in diverse experimental tasks and suggested a role in the maintenance of ‘task set’. However, this same region has been linked to other forms of processing, namely ‘known uncertainty’ or risk processing [93], and adjudicating between competing options (for review a discussion see [59,94]). These alternative explanations would be equally plausible explanations for coding outcome probability for familiar, but not novel, CS. 

## 5. Conclusions and Limitation

Several shortcomings of the current study need further mentioning. Firstly, one of the most important drawbacks of the current study is the limited number of participants. Although the total number of participants in the current study and the significance levels are adequate according to previously reported standards [54,95,96], more recent studies suggest that an increased number of participants of up to 100 dramatically increases the replicability of the fMRI studies [97]. This is especially important for the PPI analysis, because only 14 participants with peak activations in those regions were included in the analysis. Secondly, although participants reported not being able to speak Chinese, and being unfamiliar with the stimulus form, possible previous exposure to those fonts through the media might have influenced their perceptual familiarity. Thirdly, as discussed earlier, there are other behavioral criteria’s that might indicate automatization during decision making [20,24,34,40], and with a different criteria it is possible to show that savings in reaction time may continue after a thousand trials [98]. Therefore, caution is needed in interpreting the findings.

Finally, unlike standard functional connectivity analysis or effective connectivity analysis, PPI (psychophysiological interactions) analysis shows the correlation between two brain areas based on the task. In PPI analysis, unlike default mode functional coupling, the connectivity between DLPFC (and other regions for novel > familiar) and MPFC (and other regions for familiar > novel) are inherently involved in the decision-making task because the correlation is time-locked to behavioral signature. However, for future studies, we believe that it is important to perform effective connectivity analyses (such as dynamic causal modeling) to provide clear evidence of the causal shift of activation from anterior to posterior regions during the automatization of decision-making behavior.

## Figures and Tables

**Table 1:** ANOVA for the conjunction analysis: all conditional stimuli (CS) (novel and familiar) vs. baseline ^1^.

Cluster Size (Voxels)	Montreal Neurological Institute (MNI) x, y, z {mm}	F-Value (Peak)	Side	Regions of Activation
				*Frontal Cortex*
355	−6, 23, −14	26.79	L	Medial Orbito Frontal
168	−27, 32, 52	22.93	L	Middle/Superior Frontal Gyri
62	−33, −1, 58	22.95	L	Middle Frontal Gyrus
29	27, 50, −8	20.00	R	Middle/Superior Frontal Gyri
				*Temporal/Temporoparietal Cortex*
404	−51, −73, 25	49.50	L	Temporal Gyrus
	−60, −58, 22	39.66	L	Angular Gyrus
174	57, −64, 28	41.10	R	Angular Gyrus
	−3, 29, −2	14.32	L	Anterior Cingulate
143	−54, 5, −20	26.73	L	Middle Temporal Gyrus
	−45, 20, −29	18.64	L	Superior Frontal Gyrus
146	−63, −37, 1	25.92	L	Middle Temporal Gyrus
77	54, −1, −17	22.79	R	Middle Temporal Gyrus
				*Parietal Cortex*
15859	24, −67, 40	121.39	R	Precuneus
248	−6, −52, 34	24.76	L	Precuneus
68	60, −13, 25	33.07	R	Postcentral gyrus
65	45, −25, 64	28.72	R	Postcentral gyrus
46	−12, 62, 19	18.06	L	Precuneus
11	−12, −40, 67	14.63	L	Postcentral gyrus
				*Other*
150	0, −28, 28	51.93		Cingulate Gyrus
24	−30, −85, −29	23.56	L	Cerebellum
20	27, −88, −29	16.39	R	Cerebellum

^1^ All peak voxels significant at *p* < 0.05, FWE corrected.

**Table 2:** ANOVA for the conjunction analysis: main effect of uncertainty and uncertainty × novelty interaction ^1^.

Cluster Size (Voxels)	MNI x, y, z {mm}	F−Value (Peak)	Side	Regions of Activation
Main Effect of Uncertainty				
158	3, 26, 46	14.22	R	Superior Medial Gyrus (BA8)
62	33, 23, −5	17.76	R	Inferior Frontal Gyrus
87	−30, 23, 4	17.72	L	Inferior Frontal Gyrus
*Striatum*				
95	−30, −7, −2	14.27	L	Putamen
47	27, −1, −2	10.77	R	Putamen
10	12,20,1	8.88	R	Caudate
*Temporal/Temporoparietal*				
139	−54, −58, 22	11.2	L	Middle Temporal Gyrus
	−45, −73, 28	9.52	L	Angular Gyrus
30	−45, −55, −8	13.24	L	Inferior Temporal Gyrus
16	−51, 35, 28	10.75	L	Middle Temporal Gyrus
14	−15, −34, 43	10.46	R	Middle Temporal Gyrus
*Parietal*				
226	−3, −55, 31	12.37	L	Precuneus
	−6, −43, 31	11.43	L	Posterior Cingulate Cortex
13	−45, −67, 43	9.37	L	Angular Gyrus
20	54, −58, 28	9.11	R	Angular Gyrus
16	−39, −52, 52	9.34	L	Inferior Parietal Lobule
Uncertainty × Novelty Interaction				
*Frontal Cortex*				
25	12, 20, 52	9.84	R	Superior/Medial (BA8)
23	−18, 23, 28	12.13	L	Superior Frontal Gyrus
16	42, 14, 46	10.6	R	Middle Frontal Gyrus (BA8)
*Parietal and Posterior Cingulate Cortex*				
48	−39, −40, 31	19.18	L	Inferior Parietal Lobule (BA40)
16	39, −46, 37	9.19	R	Inferior Parietal Lobule (BA40)
43	−15, −55, 34	11.67	L	Precuneus
	−18, −52, 25	9.7	L	Cingulate Gyrus
22	12, −76, 46	9.46	R	Precuneus
119	21, −46, 28	12.96	R	Cingulate Gyrus
117	9, 38, 34	16.79	R	Middle Cingulate Cortex
	21, −64, 40	9.31	R	Right Precuneus
*Occipital Cortex*				
124	−27, −91, 19	11.01	L	Middle Occipital Gyrus
78	−45, −85, −2	12.4	L	Middle/Inferior Occipital Gyri
25	21, −82, 10	10.28	R	Calcarine Gyrus (BA 17)
41	33, −67, 1	9.85	R	Middle Occipital Gyrus

^1^ All peak voxels were significant at *p* < 0.001, uncorrected (*k* = 10 voxels). *K* = a minimum cluster size.

**Table 3:** Subtraction analysis: Novel CS > Familiar CS ^1^.

Cluster Size (Voxel)	MNI x, y, z {mm}	T-Value (Peak)	Side	Regions of Activation
				*Frontal Cortex*
75	42, 32, 19	4.65	R	Middle Frontal Gyrus (BA46)
14	−42, 29, 19	3.75	L	Middle Frontal Gyrus (BA46)
70	45, 8, 37	4.61	R	Precentral Gyrus
67	−42, −1, 37	4.27	L	Precentral Gyrus
28	24, −1, 49	4.21	R	Premotor Area (BA6)
				*Temporal Cortex*
312	48. −58, −11	5.75	R	Inferior Temporal & Fusiform Gyri
	−45, −58, −11	4.79	L	Inferior Temporal Gyrus
	48, 0.79, 0.5	3.44	R	Inferior Occipital Gyrus
				*Parieto0ccipitalCortex*
557	33, −49, 52	4.65	R	Inferior Parietal Lobule
	−24, −73, 46	5.48	L	Superior Parietal Lobule
	27, −70, 43	5.93	R	Sup/Mid/Inf Occipital Gyri
	39, −79, 16	4.61	R	Middle Occipital Gyrus
	27, −70, 43	5.93	R	Sup/Mid/Inf Occipital Gyri
				*Insular Cortex*
29	−27, 23, −2	4.8	L	Insula
				*Occipital Cortex*
809	45, −79, −8	5.7	L	Inferior Occipital Gyrus

^1^ All peak voxels were significant at *p* < 0.001, uncorrected (*k* = 10 voxels). *K* = minimum cluster size.

**Table 4:** Subtraction analysis: Familiar CS > Novel CS ^1^.

Cluster Size (Voxel)	MNI x, y, z {mm}	T-Value (Peak)	Side	Regions of Activation
				*Frontal*
13	−12, 56, 4	3.56	L	Medial Frontal Gyrus (BA10)
12	12, 47, −5	3.54	R	Medial Frontal Gyrus (BA 10/BA32)
				*Temporal*
28	−45, −79, 34	4.03	L	Middle Temporal Gyrus
	−51, −76, 28	3.86	L	Middle Temporal Gyrus
				*Parietal and Cingulate*
12	−15, −52, 37	3.99	L	Precuneus
19	66, −43, 31	3.52	R	Supramarginal Gyrus
19	18, 2, 28	4.14	R	Cingulate Gyrus
23	9, −19, 43	3.77	R	Middle Cingulate Cortex
30	12, 91, 34	3.7	R	Superior Occipital Gyrus
	9, −91, 19	3.51	R	Cuneus (BA7)
14	−9, −76,31	3.4	L	Cuneus (BA7)
				*Occipital Cortex*
30	12, 91, 34	3.7	R	Superior Occipital Gyrus
13	15, 43, −5	3.66	R	Lingual Gyrus
				*Other*
44	3, 20, 16	4.25	R	Corpus Callosum
	9, 11, 22	3.44	L	Corpus Collosum

^1^ All peak voxels were significant at *p* < 0.001, uncorrected (*k* = 10 voxels). *K* = minimum cluster size.

**Table 5:** PPI analysis for Novel CS > Familiar CS ^1^.

Cluster Size (Voxel)	MNI x, y, z {mm}	*T*−Value (Peak)	Side	Regions of Activation
				*Striatum*
30	9, 11, −2	4.5	R	Caudate
	18, 5, −11	4.37	R	Putamen
	30, 2, −14	4.06	R	Putamen
13	−33, −25, −8	4.99	L	Caudate Tail covering hippocampus
				*Frontal*
293	−3, 5, 58	6.69	L	Supplementary Motor Area
	9, 5, 28	4.69	R	Corpus Callosum
	−6, 20, 34	4.63	L	Cingulate Gyrus
87	45, 8, 28	5.79	R	Inferior Frontal Gyrus
48	−45,8,28	4.92	L	Inferior Frontal Gyrus
17	33, −1, 61	4.44	R	Middle Frontal Gyrus
10	39, 44, 22	4.18	R	Middle Frontal Gyrus
38	−48, −19, 37	4.48	L	Precentral Gyrus
30	18, −31, 64	4.37	R	Precentral Gyrus (BA 4)
38	−48, −19, 37	4.48	L	Precentral Gyrus
				*Temporal*
534	33, −16, 43	6.54	R	Middle Temporal Gyrus
	48, −67, 4	5.36	R	BA 37
13	36, 2, −26	5.89	R	Superior Temporal/R Amygdala
				*Insula*
189	−27, 17, 22	5.62	L	Insula
22	30, 20, 4	5.02	R	Insula
				*Parietal*
65	21, −61, 58	5.84	R	Superior Parietal Lobule/BA 7
13	−30, −34, 43	4.22	L	Parietal Lobe
67	−24, −91, 19	4.76	L	Cuneus
	−6, −97, 16	4.28	L	BA 18 (Occipital)
				*Occipital/Temporal*
314	−42, 52, −5	7.57	L	Occipital Lobe
	−42, 64, 4	5.65	L	Middle Temporal Gyrus
	−36, −40, 7	552	L	Superior Temporal Gyrus
162	−15, −88, −11	6.8	L	Occipital Lobe
	−24, −88, −11	6.34	L	Lingual Gyrus
60	33, −76, −11	5.69	R	Occipital Lobe
				*Limbic*
20	−12, −7, 34	4.71	L	Cingulate Gyrus
20	−12, −34, 34	4.5	L	Cingulate Gyrus
				*Other*
23	−48, −58, −23	7.39	L	Cerebellum Posterior Lobe
16	−21, −64, −44	4.84	L	Cerebellum Posterior Lobe
14	30, −64, −47	5.05	R	Cerebellum Posterior Lobe
44	−18, −61, −14	4.73	L	Cerebellum
10	21, −13, −2	4.36	R	Thalamus

^1^ All peak voxels were significant at *p* < 0.001, uncorrected (*k* = 10 voxels). *K* = minimum cluster size.

**Table 6:** PPI analysis for Familiar CS > Novel CS ^1^.

Cluster Size (Voxel)	MNI x, y, z {mm}	*T*-Value (Peak)	Side	Regions of Activation
				*Striatum*
88	−36, −4, 7	8.31	L	Putamen
31	30, 17, 4	6.33	R	Putamen
				*Temporal Cortex*
10	51, −58, 4	5.35	R	Middle Temporal Gyrus
				*Parietal Cortex*
23	30, −61, 43	7.03	R	Precuneus
20	−33, 0.52, 46	6.69	L	Parietal Lobe
24	33, −52, 55	6.44	R	Inferior Parietal Lobule
38	51, −25, 52	6.06	R	Postcentral Gyrus
				*Occipital Cortex*
78	49, 48, 4	7.03	L	Occipital Lobe/Inf Occ Gyrus
16	36, 48, 13	5.35	R	Occipital Lobe
				*Limbic*
31	−3, 14, 40	6.64	L	Cingulate Gyrus (BA32)
	6, 14, 43	5.07	R	Cingulate Gyrus

^1^ All peak voxels were significant at *p* < 0.001, uncorrected (*k* = 10 voxels). *K* = minimum cluster size.
